# Novel tetrazole and cyanamide derivatives as inhibitors of cyclooxygenase-2 enzyme: design, synthesis, anti-inflammatory evaluation, ulcerogenic liability and docking study

**DOI:** 10.1080/14756366.2017.1326110

**Published:** 2017-06-06

**Authors:** Phoebe F. Lamie, John N. Philoppes, Amany A. Azouz, Nesreen M. Safwat

**Affiliations:** a Department of Pharmaceutical Organic Chemistry, Beni-Suef University, Beni-Suef, Egypt;; b Department of Pharmacology and Toxicology, Faculty of Pharmacy, Beni-Suef University, Beni-Suef, Egypt;; c Pathology Department, Faculty of Veterinary Medicine, Beni-Suef University, Beni-Suef, Egypt

**Keywords:** Tetrazole, cyanamide, anti-inflammatory, ulcerogenicity, histopathology

## Abstract

Nineteen new compounds containing tetrazole and/or cyanamide moiety have been designed and synthesised. Their structures were confirmed using spectroscopic methods and elemental analyses. Anti-inflammatory activity for all the synthesised compounds was evaluated *in vivo*. The most active compounds **4c**, **5a**, **5d–f**, **8a** and **b** and **9a** and **b** were further investigated for their ulcerogenic liability and analgesic activity. Pyrazoline derivatives **9b** and **8b** bearing trimethoxyphenyl part and SO_2_NH_2_ or SO_2_Me pharmacophore showed equal or nearly the same ulcerogenic liability (UI: 0.5, 0.75, respectively), to celecoxib (UI: 0.50). Most of tested compounds showed potent central and/or peripheral analgesic activities. Histopathological investigations were done to evaluate test compounds effect on rat's gastric tissue. The obtained results were in consistent with the *in vitro* data on COX evaluation. Docking study was also done for all the target compounds inside COX-2-active site.

## Introduction

Tetrazole represents an important class of heterocyclic compounds for biological and pharmacological applications. The tetrazole ring is a bioisostere of the carboxylic acid (–COOH) group so they have close p*K*_a_ values, also, it has similar planar skeleton structure and nitrogen-rich multi-electron conjugated system^1–4^. At the same time, it does not have the acidic –COOH properties, and so, it helps in decreasing toxic properties of drugs^5^. 1,2,3,4-Tetrazole derivatives possess potent pharmacological activities such as antimicrobial^6–9^, antihypertensive^10^^,^^11^, anticonvulsant^12^^,^^13^, anticancer^14^^,^^15^, analgesic^4^^,^^16^, antiulcer^17^ and anti-inflammatory^3^^,^^18–22^.

On the other hand, pyridine scaffold^23–25^ was the base of many bioactive molecules especially anti-inflammatory agents. Moreover, pyrazoline ring^26–29^, hydrazone functionality^30^ and chalcones^31–33^ are attractive motifs for anti-inflammatory drugs.

**Figure 1.**
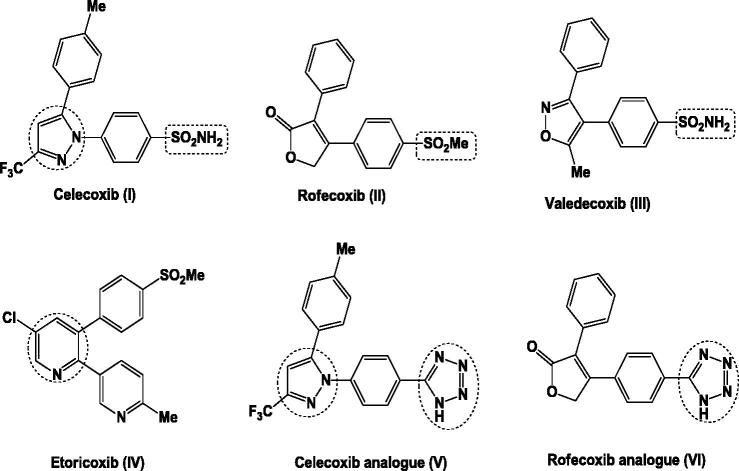
General structure of some known selective COX-2 inhibitors.

Non-steroidal anti-inflammatory drugs (NSAIDs) are of great importance in the treatment of inflammation and pain. They act by inhibiting cyclooxygenase enzymes (COXs) – a membrane-bound haeme protein – that control the conversion of arachidonic acid to prostaglandins and thromboxanes. Two distinct isoforms of COX enzyme are present, a constitutive form (COX-1), associated with several side effects such as haemorrhagia and gastrointestinal (GI) ulceration, and an inducible form (COX-2) which is different in the regulation and tissue distribution from COX-1^34–36^. In 2012, Al-Hourani et al. reported a novel COX-1 splice variant termed as COX-3^37^. COX-1 and COX-2 use identical catalytic mechanism to catalyse the same reactions by sharing the same substrates and producing the same products. They are very similar in their protein tertiary conformation as demonstrated from their X-ray crystal structures^38^. These similarities in the structure of both COX isoforms represent a great challenge for the development of selective COX-2 inhibitors. The space of selectivity pocket is the main difference between the two isoforms. It is reduced in COX-1 due to the presence of Ile523 rather than Val523 in COX-2. Conformational change is occurred as a result of the presence of Val523 in COX-2, leading to the formation of additional hydrophobic secondary internal pocket protruding off the primary binding site^39^. A large number of compounds have been synthesised and evaluated for their selective COX-2 inhibitory activity. Celecoxib (I), rofecoxib (II), valedecoxib (III) and etoricoxib (IV) are the most common approved selective COX-2 inhibitors. However, inhibition of COX-2 reduces urinary sodium excretion leading to increase in blood pressure as well as myocardial infarction, that is why rofecoxib and valdecoxib had been withdrawn from the market (Figure 1)^40–42^.

Until now, celecoxib has been one of the most popular selective COX-2 drugs. So, there is still a need for designing and developing new effective anti-inflammatory drugs with improved safety profiles.

New water-soluble, parentral COX-2 inhibitors containing tetrazole moiety in their structures, such as celecoxib and rofecoxib analogues (V, VI, respectively) were reported to have *in vivo* anti-inflammatory activity and exhibited a high COX-2/COX-1 selectivity (SI = 2.16, 2.11, sequentially) when compared to the reference celecoxib (SI = 1.68). Both of them lacked the side effect of gastric ulceration (Figure 1)^43^.

The majority of selective COX-2 inhibitors are of five-membered heterocyclic ring as pyrazole in celecoxib (I), or attached to pyridine six-membered ring as in etoricoxib (IV)^44^. Moreover, pyrazoline present in antipyrin – the first pyrazoline derivative used in the treatment of pain and inflammation – and several related analogues as in felcobuzone, morazone and ramifenazone are also available as NSAIDs^45^.

Furthermore, different important selective COX-2 pharmacophores such as aminosulphonyl and methylsulphonyl groups are essential for potent and selective anti-inflammatory activity^40^^,^^41^.

**Figure 2.**
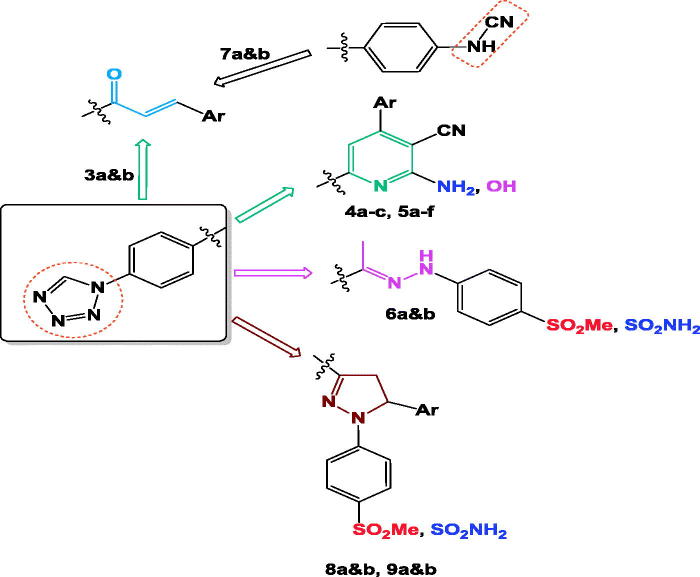
Design for the newly synthesised compounds **3a&b**: **9a&b**.

In view of the above**-**mentioned facts and as a continuation of our previous work on the development of selective COX-2 inhibitors^36^^,^^40^^,^^46^, four groups of compounds have been synthesised: **(**i) chalcone derivatives **3a** and **b** and **7a** and **b**, **(**ii) pyridine containing compounds **4a–c** and **5a–f**, (iii) hydrazone of methylsulphonyl and aminosulphonyl derivatives **6a** and **b** and (iv) five-membered pyrazoline ring**-**bearing methylsulphonyl **8a** and **b** group or aminosulphonyl **9a** and **b** moiety, (Figure 2). Most of the prepared compounds were linked to tetrazole ring aiming to generate novel molecular templates for safe anti-inflammatory agents. The synthesised compounds were subjected to *in vitro* evaluation as (COX-1/COX-2) inhibitors and *in vivo* (AI) activity. Analgesic activity and ulcer index (UI) have also been studied. Moreover, the effect of the most active synthesised compounds on rat’s gastric tissue was evaluated using histopathological study. Finally, docking study was performed on COX-2 enzyme to explore the possible binding mode of the designed compounds inside the enzyme.

## Experimental section

### Chemistry

Melting points were determined using a Griffin apparatus and were uncorrected. IR spectra were recorded on a Shimadzu IR-435 spectrophotometer using KBr discs and values were represented in cm^−1^. ^1^H NMR and ^13 ^C NMR (DEPT-Q) were carried out on Bruker apparatus at 400 MHz for ^1^H NMR and 100 MHz for ^13 ^C NMR spectrophotometer, (Faculty of Pharmacy, Beni-Suef University, Beni-Suef, Egypt), in DMSO-d_6_, D_2_O using TMS as an internal standard and chemical shifts were recorded in ppm on *δ* scale using DMSO-d_6_ (2.5) as a solvent. Coupling constant (*J*) values were estimated in Hertz (Hz). Splitting patterns are designated as follows: *s*, singlet; *d*, doublet, *t*, triplet; *q*, quartet; dd, doublet of doublet; *m*, multiplet. The electron impact (EI) mass spectra were recorded on Hewlett Packard 5988 spectrometer (Palo Alto, CA). Microanalysis was performed for C, H, N on Perkin-Elmer 2400 at the Microanalytical center, Cairo University, Egypt and was within ±0.4% of theoretical values. Analytical thin-layer chromatography (TLC): pre-coated plastic sheets, 0.2 mm silica gel with UV indicator (Macherey-Nagel) was employed routinely to follow the course of reactions and to check the purity of products. All other reagents, solvents and compound **1** were purchased from the Aldrich Chemical Company (Milwaukee, WI), were used without further purification.

### General procedure for synthesis of compounds 3a and b

A mixture of **2** (2 g, 0.01 mol) and KOH (0.56 g, 0.01 mol) in absolute ethanol (20 mL), was stirred at room temperature for 30 min. Then, the corresponding aldehyde (0.01 mol) was added and the reaction mixture was stirred at room temperature for about 10–12 h. The solid separated was filtered, dried and crystallised from DMF/ethanol (1:2) to give **3a** and **b**.

#### (ZE)-1-[4-(1H-Tetrazol-1-yl)phenyl]-3-(3,4-dimethoxyphenyl)prop-2-en-1-one (3a)

Yellow solid; (1.53 g, 86%) yield; mp 214–216 °C; IR (KBr) 1674 (C = O) cm^−1^; ^1^H NMR (400 MHz, DMSO-d*_6_*) *δ*: 3.83 (*s*, 3H, OCH_3_), 3.88 (*s*, 3H, OCH_3_), 7.05 (d, *J=* 8 Hz, 1H, dimethoxyphenyl H-5), 7.45 (d, *J=* 8 Hz, 1H, dimethoxyphenyl H-6), 7.58 (*s*, 1H, dimethoxyphenyl H-2), 7.77 (d, *J=* 15.2 Hz, 1H, COCH = CH), 7.90 (d, *J* = =15.2, 1H, COCH = CH), 8.12 (d, *J=* 8 Hz, 2H, phenyl H-3, H-5), 8.42 (d, *J* = 8 Hz, 2H, phenyl H-2, H-6), 10.27 (*s*, 1H, tetrazole H); ^13 ^C NMR (100 MHz, DMSO-d_6_) *δ*: 56.63 (OCH_3_), 60.53 (OCH_3_), 107.16 (dimethoxyphenyl C-2), 107.78 (dimethoxyphenyl C-5), 121.40 (–CO–CH = CH–), 121.46 (dimethoxyphenyl C-6), 121.55 (phenyl C-3, C-5), 130.52 (dimethoxyphenyl C-1), 130.93 (phenyl C-2, C-6), 137.17 (phenyl C-1), 138.59 (phenyl C-4), 142.90 (tetrazole C-5), 145.87 (–CO–CH = CH–), 153.58 (dimethoxyphenyl C-3), 188.60 (C = O); EIMS (*m*/*z*) 336 (*M*^+^**^.^**, 31.93%), 145 (100%). Anal. Calcd for C_18_H_16_N_4_O_3_: C, 64.28; H, 4.79; N, 16.66. Found: C, 64.37; H, 4.65; N, 16.51.

#### (ZE)-1-[4-(1H-Tetrazol-1-yl)phenyl]-3-(3,4,5-trimethoxyphenyl)prop-2-en-1-one (3b)

Yellow solid; (1.53 g, 79%) yield; mp 215–217 °C; IR (KBr) 1663 (C = O) cm^−1^; ^1^H NMR (400 MHz, DMSO-d*_6_*) *δ*: 3.73 (*s*, 3H, *p*-OCH_3_), 3.88 (*s*, 6H, 2 *m*-OCH_3_), 7.28 (*s*, 2H, trimethoxyphenyl H-2, H-6), 7.77 (d, *J* = 15.2 Hz, 1H, COCH = CH), 7.95 (d, *J* = 15.2, 1H, COCH = CH), 8.16 (d, *J* = 8.4 Hz, 2H, phenyl H-3, H-5), 8.42 (d, *J* = 8.4 Hz, 2H, phenyl H-2, H-6), 10.27 (*s*, 1H, tetrazole H); ^13 ^C NMR (100 MHz, DMSO-d_6_) *δ*: 56.65 (2 *m*-OCH_3_), 60.64 (*p*-OCH_3_), 107.25 (trimethoxyphenyl C-2, C-6), 121.41 (–CO–CH = CH–), 121.52 (phenyl C-3, C-5), 130.54 (trimethoxyphenyl C-1), 130.93 (phenyl C-2, C-6), 137.23 (trimethoxyphenyl C-4), 138.60 (phenyl C-1), 140.46 (phenyl C-4), 142.97 (tetrazole C-5), 145.86 (–CO–CH = CH–), 153.60 (trimethoxyphenyl C-3, C-5), 188.49 (C = O); Anal. Calcd for C_19_H_18_N_4_O_4_: C, 62.29; H, 4.95; N, 15.29. Found: C, 62.15; H, 5.03; N, 15.32.

### General procedure for synthesis of 4a–c

#### Method A

To a mixture of **2** (2 , 0.01 mol) and the appropriate aromatic aldehyde (0.01 mol) in absolute ethanol (20 mL), malononitrile (0.66 g, 0.01 mol) and ammonium acetate (1.54 g, 0.02 mol) were added. The reaction mixture was refluxed for 20 h. The reaction mixture was cooled then poured into crushed ice. The obtained solid was filtered off, dried and crystallised from 95% ethanol to give **4a** and **b**.

#### Method B (for preparation of 4c)

An ethanolic mixture of chalcone **3a** (3.36 g, 0.01 mol) and malononitrile (0.66 g, 0.01 mol) in the presence of ammonium acetate (1.54 g, 0.02 mol) was refluxed for 8–10 h. After cooling, the obtained solid was filtered off, dried and crystallised from 95% ethanol to give **4c**.

#### 6-[4-(1H-Tetrazol-1-yl)phenyl]-2-amino-4-phenylnicotinonitrile (4a)

Yellow solid; method A; (1.62 g, 45%) yield; mp 238–240 °C; IR (KBr) 3229, 3117 (NH_2_), 2210 (C≡N) cm^−1^; ^1^H NMR (400 MHz, DMSO-d_6_) *δ*: 6.93–6.94 (*m*, 3H, phenylnicotinonitrile H-3, H-4, H-5), 7.54–7.66 (*m*, 5H, phenylnicotinonitrile H-2, H-6, pyridine H-5, NH_2_, D_2_O exchangeable), 7.95 (d, 2H, *J* = 8.4 Hz, phenyl H-3, H-5), 8.08 (d, 2H, *J* = 8.4 Hz, phenyl H-2, H-6), 10.20 (*s*, 1H, tetrazole H-5); ^13 ^C NMR (100 MHz, DMSO-d_6_) *δ*: 97.05 (pyridinecarbonitrile C-3), 107.93 (pyridinecarbonitrile C-5), 116.81 (C≡N), 121.61 (phenyl-6-yl C-2, C-6), 128.76 (phenyl-4-yl C-2, C-6), 129.31 (phenyl-4-yl C-3, C-5), 130.08 (phenyl-6-yl C-3, C-5), 130.98 (phenyl-4-yl C-4), 133.93 (phenyl-4-yl C-4), 135.83 (phenyl-6-yl C-1), 136.35 (phenyl-4-yl C-1), 142.79 (tetrazole C-5), 160.12 (pyridine C-4), 162.71 (pyridine C-6), 162.87 (pyridine C-2); EIMS (*m*/*z*) 339 (M^+^**^.^**, 0.84%), 335 (100%). Anal. Calcd for C_19_H_13_N_7_: C, 67.25; H, 3.86; N, 28.89. Found: C, 67.41; H, 3.92; N, 28.74.

#### 6′-[4-(1H-Tetrazol-1-yl)phenyl]-2′-amino-[3,4′-bipyridine]-3′-carbonitrile (4b)

Yellow solid; method A; (2.21 g, 57%) yield; mp 238–240 °C; IR (KBr) 3352, 3136 (NH_2_), 2210 (C≡N) cm^−1^; ^1^H NMR (400 MHz, DMSO-d_6_) *δ*: 6.99 (*s*, 2H, NH_2_, D_2_O exchangeable), 7.03 (*s*, 1H, pyridinecarbonitrile H-5), 7.59 (dd, *J* = 4.8 Hz, 8 Hz, 1H, pyridine H-5), 7.96 (d, *J* = 8.4 Hz, 2 H, phenyl H-3, H-5), 8.09–8.13 (*m*, 3H, phenyl H-2, H-6 and pyridine H-6), 8.73 (d, *J*= 4.8 Hz, 1H, pyridine H-4), 8.86 (*s*, 1H, pyridine H-2), 10.20 (*s*, 1H, tetrazole H-5); ^13 ^C NMR (100 MHz, DMSO-d_6_) *δ*: 95.09 (pyridinecarbonitrile C-3), 112.99 (pyridinecarbonitrile C-5), 116.24 (C≡N), 121.85 (phenyl C-2, C-6), 124.00 (pyridine C-5), 130.94 (phenyl C-3, C-5), 134.74 (phenyl C-4), 137.16 (phenyl C-1), 138.75 (pyridine C-6), 139.65 (pyridine C-1), 142.68 (tetrazole C-5), 149.07 (pyridine C-4), 150.91 (pyridine C-2), 154.46 (pyridinecarbonitrile C-6), 157.05 (pyridinecarbonitrile C-4), 162.14 (pyridinecarbonitrile C-2); Anal. Calcd for C_18_H_12_N_8_: C, 63.52; H, 3.55; N, 32.92. Found: C, 63.35; H, 3.42; N, 32.80.

#### 6-[4-(1H-Tetrazol-1-yl)phenyl]-2-amino-4-(3,4-dimethoxyphenyl)nicotinonitrile (4c)

Yellow solid; method B; (2.06 g, 61%) yield; mp 230–232 °C; IR (KBr) 3210, 3121 (NH_2_), 2207 (C≡N) cm^−1^; ^1^H NMR (400 MHz, DMSO-d_6_) *δ*: 3.82 (*s*, 6H, 2OCH_3_), 6.95 (*s*, 1H, dimethoxyphenyl H-2), 7.01–7.06 (*m*, 2H, dimethoxyphenyl H-5, H-6), 7.20 (*s*, 2H, NH_2_, D_2_O exchangeable), 7.78–7.88 (*m*, 3H, phenyl H-3, H-5, pyridine H-5), 8.15 (d, *J* = 8.4 Hz, 2H, phenyl H-2, H-6), 10.21 (*s*, 1H, tetrazole H-5); ^13 ^C NMR (100 MHz, DMSO-d_6_) *δ*: 56.03 (OCH_3_), 56.35 (OCH_3_), 90.87 (pyridine C-3), 109.17 (pyridine C-5), 110.95 (dimethoxyphenyl C-5), 112.19 (dimethoxyphenyl C-2), 114.64 (C≡N), 117.59 (dimethoxyphenyl C-6), 121.50 (phenyl C-2, C-6), 130.59 (dimethoxyphenyl C-1), 130.89 (phenyl C-3, C-5), 134.53 (phenyl C-4), 139.64 (phenyl C-1), 142.85 (tetrazole C-5), 149.10 (dimethoxy phenyl C-3), 151.08 (dimethoxyphenyl C-4), 154.02 (pyridine C-6), 157.00 (pyridine C-4), 161.74 (pyridine C-2); EIMS (*m/z*) 399 (M^+^**^.^**, 3.64%), 214 (100%). Anal. Calcd for C_21_H_17_N_7_O_2_: C, 63.15; H, 4.29; N, 24.55. Found: C, 63.44; H, 4.17; N, 24.38.

### General procedure for synthesis of 5a–f

#### Method A

To a mixture of **2** (2 g, 0.01 mol) and the corresponding aromatic aldehyde (0.01 mol) in absolute ethanol (20 mL), ethyl cyanoacetate (1.13 g, 0.01 mol) and ammonium acetate (1.54 g, 0.02 mol) were added. The reaction mixture was refluxed for 24 h. The reaction mixture was cooled and then poured into crushed ice. The obtained solid was filtered off, dried and crystallised from 95% ethanol to give **5a–d**.

#### Method B (for preparation of 5e and 5f)

An ethanolic mixture of the selected chalcones **3a** or **3b** (0.01 mol) and ethyl cyanoacetate (1.13 g, 0.01 mol) in the presence of ammonium acetate (1.54 g, 0.02 mol) was refluxed for 5–6 h. After cooling, the obtained solid was filtered off, dried and crystallised from 95% ethanol to give **5e** and **5f**.

#### 6-[4-(1H-Tetrazol-1-yl)phenyl]-2-oxo-4-phenyl-1,2-dihydropyridine-3-carbonitrile (5a)

Yellow solid; method A, (1.73 g, 48%) yield; mp 281–283 °C; IR (KBr) 3483 (OH), 2222 (C≡N), 1659 (C = O) cm^−1^; ^1^H NMR (400 MHz, DMSO-d_6_) *δ*: 7.02 (*s*, 1H, pyridine H-5), 7.59–7.60 (*m*, 3H, phenylnicotinonitrile H-3, H-4, H-5), 7.76–7.78 (*m*, 2H, phenylnicotinonitrile H-2, H-6), 8.11 (d, *J* = 8.8 Hz, 2H, phenyl H-3, H-5), 8.22 (d, *J* = 8.8 Hz, 2H, phenyl H-2, H-6), 10.22 (*s*, 1H, tetrazole H-5), 12.94 (*s*, 1H, OH, D_2_O exchangeable); ^13 ^C NMR (100 MHz, DMSO-d_6_) *δ*: 95.06 (pyridinecarbonitrile C-3), 116.35 (C≡N), 119.00 (pyridinecarbonitrile C-5), 121.43 (phenyl-6-yl C-2, C-6), 127.58 (phenyl-4-yl C-2, C-6), 128.87 (phenyl-4-yl C-3, C-5), 129.20 (phenyl-4-yl C-4), 130.02 (phenyl-6-yl C-3, C-5), 134.84 (phenyl-6-yl C-4), 137.82 (phenyl-6-yl C-1), 139.00 (phenyl-4-yl C-1), 142.88 (tetrazole C-5), 148.76 (pyridine C-4), 150.54 (pyridine C-6), 154.58 (pyridine C-2); Anal. Calcd for C_19_H_12_N_6_O: C, 67.05; H, 3.55; N, 24.69. Found: C, 66.89; H, 3.61; N, 24.53.

#### 6′-[4-(1H-Tetrazol-1-yl)phenyl]-2′-hydroxy-[3,4′-bipyridine]-3′-carbonitrile (5b)

Yellow solid; method A; (2.06 g, 57%) yield; mp 287–289 °C; IR (KBr) 3333 (OH), 3132 (NH), 2214 (C≡N), 1652 (C = O); ^1^H NMR (400 MHz, DMSO-d_6_) *δ*: 7.19 (*s*, 1H, pyridine carbonitrile H-5), 7.64 (dd, *J =* 8, 4.8 Hz, 1H, pyridine H-5), 8.13 (d, *J* = 8.8 Hz, 2H, phenyl H-2, H-6), 8.20–8.26 (*m*, 3H, phenyl H-3, H-5 and pyridine H-6), 8.77 (dd, *J* = 4.8, 1.2 Hz, 1H, pyridine H-4), 8.97 (*s*, 1H, pyridine H-2), 10.23 (*s*, 1H, tetrazole H), 12.91 (*s*, 1H, OH, D_2_O exchangeable); ^13 ^C NMR (100 MHz, DMSO-d_6_) *δ*: 95.14 (pyridinecarbonitrile C-3), 116.20 (C≡N), 119.05 (pyridinecarbonitrile C-5), 121.76 (phenyl C-2, C-6), 124.09 (pyridine C-5), 130.98 (phenyl C-3, C-5), 133.74 (phenyl C-4), 134.88 (phenyl C-1), 136.80 (pyridine C-6), 138.82 (pyridine C-1), 142.87 (tetrazole C-5), 147.22 (pyridinecarbonitrile C-6), 149.00 (pyridinecarbonitrile C-4), 149.25 (pyridine C-4), 150.83 (pyridine C-2), 154.53 (pyridinecarbonitrile C-2); EIMS (*m*/*z*) 341 (*M***^+.^**, 0.77%), 288 (100%). Anal. Calcd for C_18_H_11_N_7_O: C, 63.34; H, 3.25; N, 28.73. Found: C, 63.25; H, 3.13; N, 28.56.

#### 6-[4-(1H-Tetrazol-1-yl)phenyl]-4-(4-methoxyphenyl)-2-oxo-1,2-dihydropyridine-3-carbonitrile (5c)

Yellow solid; method A; (1.92 g, 49%) yield; mp > 300 °C; IR (KBr) 3483 (OH), 3117 (NH), 2222 (C≡N), 1659 (C = O) cm^−1^; ^1^H NMR (400 MHz, DMSO-d_6_) *δ*: 3.86 (*s*, 3H, OCH_3_), 6.97 (*s*, 1H, pyridine H-5), 7.14 (d, *J* = 8.8 Hz, 2H, Ar-H, *p*-methoxyphenyl H-3, H-5), 7.77 (d, *J* = 8.4 Hz, 2H, phenyl H-3, H-5), 8.00 (d, *J* = 8.8 Hz, 2H, *p*-methoxyphenyl H-2, H-6), 8.20 (d, *J* = 8.4 Hz, 2H, phenyl H-2, H-6), 10.22 (*s*, 1H, tertrazole H), 12.86 (*s*, 1H, OH, D_2_O exchangeable); ^13 ^C NMR (100 MHz, DMSO-d_6_) *δ*: 55.93 (OCH_3_), 92.01 (pyridine C-3), 105.05 (pyridine C-5), 114.77 (*p*-methoxyphenyl C-3, C-5), 115.93 (C≡N), 121.78 (phenyl C-2, C-6), 128.78 (*p*-methoxyphenyl C-1), 129.97 (phenyl C3, C-5), 131.41 (*p*-methoxyphenyl C-2, C-6) 135.24 (phenyl C-4), 137.26 (phenyl C-1), 142.91 (tetrazole C-5), 153.83 (pyridine C-6), 158.55 (pyridine C-4), 162.32 (*p*-methoxyphenyl C-4), 164.00 (pyridine C-2); Anal. Calcd for C_20_H_14_N_6_O_2_: C, 64.86; H, 3.81; N, 22.69. Found: C, 64.72; H, 4.05; N, 22.58.

#### 6-[4-(1H-Tetrazol-1-yl)phenyl]-4-[4-(dimethylamino)phenyl]-2-oxo-1,2-dihydropyridine-3-carbonitrile (5d)

Brown solid; method A; (1.67 g, 41%) yield; mp 263–265 °C; IR (KBr) 3445 (OH), 3129 (NH), 2210 (C≡N), 1705 (C = O) cm^−1^; ^1^H NMR (400 MHz, DMSO-d_6_) *δ*: 3.03 (*s*, 6H, 2CH_3_), 6.84–6.86 (*m*, 3H, dimethylaminophenyl H-3, H-5, pyridine H-5), 7.72 (d, *J* = 7.6 Hz, 2H, dimethylaminophenyl H-2, H-6), 8.10 (d, *J* = 8.4 Hz, 2H, phenyl H-3, H-5), 8.17 (d, *J* = 8.4 Hz, 2H, phenyl H-2, H-6), 10.22 (*s*, 1H, tetrazole H), 12.65 (*s*, 1H, OH, D_2_O exchangeable); ^13 ^C NMR (100 MHz, DMSO-d_6_) *δ*: 49.06 (2 NCH_3_), 93.76 (pyridine C-3), 101.07 (pyridine C-5), 112.01 (dimethylaminophenyl C-3, C-5), 117.87 (C≡N), 121.61 (phenyl C-2, C-6), 122.40 (dimethylaminophenyl C-1), 129.95 (dimethylaminophenyl C-2, C-6), 130.23 (phenyl C3, C-5), 135.69 (phenyl C-4), 138.69 (phenyl C-1), 142.82 (tetrazole C-5), 151.76 (pyridine C-6), 152.36 (pyridine C-4), 154.38 (dimethylaminophenyl C-4), 162.31 (pyridine C-2); EIMS (*m/z*) 383 (*M*^+^**^.^**, 2.85%), 144 (100%). Anal. Calcd for C_21_H_17_N_7_O: C, 65.79; H, 4.47, N, 25.57. Found: C, 65.56; H, 4.53; N, 25.41.

#### 6-[4-(1H-Tetrazol-1-yl)phenyl]-4-(3,4-dimethoxyphenyl)-2-oxo-1,2-dihydropyridine-3-carbonitrile (5e)

Yellow solid; method B; (2.28 g, 57%) yield; mp 272–274 °C; IR (KBr) 3325 (OH), 3132 (NH), 2218 (C≡N), 1686 (C = O) cm^−1^; ^1^H NMR (400 MHz, DMSO-d_6_) *δ*: 3.86 (*s*, 6H, 2 OCH_3_), 6.99 (*s*, 1H, pyridine H-5), 7.01 (d, *J* = 8 Hz, 1H, dimethoxyphenyl H-5), 7.38–7.40 (*m*, 2H, dimethoxyphenyl H-2, H-6), 8.11 (d, *J* = 8.4 Hz, 2H, phenyl H-3, H-5), 8.20 (d, *J* = 8.4 Hz, 2H, phenyl H-2, H-6), 10.23 (*s*, 1H, tetrazole H), 12.86 (*s*, 1H, OH, D_2_O exchangeable); ^13 ^C NMR (100 MHz, DMSO-d_6_) *δ*: 56.18 (OCH_3_), 56.21 (OCH_3_), 92.32 (pyridine C-3), 100.91 (pyridine C-5), 112.10 (dimethoxyphenyl C-5), 112.43 (dimethoxyphenyl C-2), 117.29 (C≡N), 121.61 (dimethoxyphenyl C-6), 122.05 (phenyl C-2, C-6), 122.40 (dimethoxyphenyl C-1), 128.43 (phenyl C-4), 130.08 (phenyl C3, C-5), 135.81 (phenyl C-1), 142.85 (tetrazole C-5), 146.05 (dimethoxyphenyl C-3), 149.09 (dimethoxyphenyl C-4), 151.29 (pyridine C-6), 153.63 (pyridine C-4), 161.67 (pyridine C-2); Anal. Calcd for C_21_H_16_N_6_O_3_: C, 62.99; H, 4.03; N, 20.99. Found: C, 63.04; H, 3.98; N, 20.79.

#### 6-[4-(1H-Tetrazol-1-yl)phenyl]-4-(3,4,5-trimethoxyphenyl)-2-oxo-1,2-dihydropyridine-3-carbonitrile (5f)

Yellow solid; method B; (2.32 g, 54%) yield; mp > 300 °C; IR (KBr) 3426 (OH), 3121 (NH), 2226 (C≡N), 1659 (C = O) cm^−1^; ^1^H NMR (400 MHz, DMSO-d_6_) *δ*: 3.76 (*s*, 3H, *p*-OCH_3_), 3.87 (*s*, 6H, 2 *m*-OCH_3_), 7.02 (*s*, 1H, pyridine H-5), 7.07 (*s*, 2H, trimethoxyphenyl H-2, H-6), 8.09 (d, *J* = 8.4 Hz, 2H, phenyl H-3, H-5), 8.22 (d, *J=* 8.4 Hz, 2H, phenyl H-2, H-6), 10.22 (*s*, 1H, tetrazole H-5), 12.94 (*s*, 1H, OH, D_2_O exchangeable); ^13 ^C NMR (100 MHz, DMSO-d_6_) *δ*: 56.65 (2OCH_3_), 60.63 (OCH_3_), 97.20 (pyridine C-3), 103.82 (pyridine C-5), 106.60 (trimethoxyphenyl C-2, C-6), 115.19 (C≡N), 121.59 (phenyl C-2, C-6), 129.98 (phenyl C3, C-5), 131.89 (phenyl C-4), 132.75 (trimethoxyphenyl C-1), 135.67 (phenyl C-1), 140.84 (trimethoxyphenyl C-4), 142.84 (tetrazole C-5), 150.29 (pyridine C-6), 153.34 (pyridine C-4), 154.01 (trimethoxyphenyl C-3, C-5), 159.75 (pyridine C-2); EIMS (*m*/*z*) 431 (M + 1, 2.95%), 430 (*M*^+^**^.^**, 10.09%), 136 (100%). Anal. Calcd for C_22_H_18_N_6_O_4_: C, 61.39; H, 4.22; N, 19.53. Found: C, 61.27; H, 4.29; N, 19.47.

### General procedure for preparation of 6a and b

A mixture of acetophenone derivative **2** (1.88 g, 0.01 mol) and *p*-substituted sulphonylphenylhydrazine hydrochloride derivative (0.015 mol) in absolute ethanol (20 mL) was heated under reflux for 6–8 h (monitored by TLC). The obtained solid on hot was filtered, dried and crystalised from 95% ethanol to give **6a**&**b**.

#### (*ZE*)-1-{4-[1-(2-(4-Methanesulphonylphenyl)hydrazono)ethyl]phe-nyl}-1*H*-tetrazole (6a)

Yellow solid; (1.53 g, 43%) yield; mp 258–260 °C; IR (KBr) 3440 (NH), 1277, 1126 (SO_2_) cm^−1^; ^1^H NMR (400 MHz, DMSO-d_6_) *δ*: 2.37 (*s*, 3H, CH_3_), 3.13 (*s*, 3H, SO_2_CH_3_), 7.44 (d, *J* = 8.8 Hz, 2H, methanesulphonylphenyl H-3, H-5), 7.77 (d, *J* = 8.8 Hz, methanesulphonylphenyl H-2, H-6), 7.96 (d, *J* = 8.8 Hz, 2H, phenyl H-3, H-5), 8.08 (d, *J* = 8.8 Hz, 2H, phenyl H-2, H-6), 10.05 (*s*, 1H, NH, D_2_O exchangeable), 10.15 (*s*, 1H, tetrazole H); ^13 ^C NMR (100 MHz, DMSO-d_6_) *δ*: 13.68 (CH_3_), 44.71 (SO_2_CH_3_), 113.01 (methanesulphonylphenyl C-2, C-6), 121.42 (phenyl C-3, C-5), 127.49 (methanesulphonylphenyl C-3, C-5), 129.17 (phenyl C-2, C-6), 130.48 (methanesulphonylphenyl C-4), 133.60 (phenyl C-4), 140.25 (phenyl C-1), 142.62 (tetrazole C), 143.11 (methanesulphonylphenyl C-1), 150.11 (C = N-NH); EIMS (*m*/*z*) 356 (*M*^+^**^.^**, 5.59%), 158 (100%). Anal. Calcd for C_16_H_16_N_6_O_2_S: C, 53.92; H, 4.52; N, 23.58. Found: C, 54.08; H, 4.35; N, 23.71.

#### (*ZE*)-4-{2-[1–(4-(1*H*-tetrazol)-1-yl)phenyl)ethylidene]hydrazinyl}benzenesulphonamide (6b)

Yellow solid; (1.74 g, 49%) yield; mp 255–257 °C; IR (KBr) 3449–3109 (NH & NH_2_), 1273, 1157 (SO_2_) cm^−1^; ^1^H NMR (400 MHz, DMSO-d_6_) *δ*: 2.35 (*s*, 3H, CH_3_), 7.11(*s*, 2H, NH_2_, D_2_O exchangeable), 7.38 (d, *J* = 8.8 Hz, 2H, benzenesulphonamide H-3, H-5), 7.70 (d, *J* = 8.8 Hz, 2H, benzenesulphonamide H-2, H-6), 7.95 (d, *J* = 8.4 Hz, 2H, phenyl H-3, H-5), 8.08 (d, *J* = 8.4 Hz, 2H, phenyl H-2, H-6), 9.90 (*s*, 1H, NH, D_2_O exchangeable), 10.15 (*s*, 1H, tetrazole H); ^13 ^C NMR (100 MHz, DMSO-d_6_) *δ*: 13.48 (CH_3_), 112.71 (benzenesulphonamide C-2, C-6), 121.44 (phenyl C-3, C-5), 127.40 (benzenesulphonamide C-3, C-5), 127.73 (phenyl C-2, C-6), 133.39 (benzenesulphonamide C-4), 134.28 (phenyl C-4), 140.38 (phenyl C-1), 142.29 (benzenesulphonamide C-1), 142.54 (tetrazole C), 148.74 (C = N-NH); Anal. Calcd for C_15_H_15_N_7_O_2_S: C, 50.41; H, 4.23; N, 27.43. Found: C, 50.37; H, 4.15; N, 27.17.

### General procedure for synthesis of 7a and b

To a solution of **3a** or **3b** (0.01 mol) in absolute ethanol (30 mL), urea or thiourea (0.01 mol) and KOH (0.56 g, 0.01 mol) were added. The reaction mixture was heated under reflux for 10–12 h (monitored by TLC). The solid obtained was filtered, dried and crystallised from 95% ethanol to afford **7a**&**b**.

#### (*ZE*)-*N*-{4-[3–(3,4-Dimethoxyphenyl)acryloyl]phenyl}cyanamide (7a)

Orange solid; (1.23 g, 40%) yield; mp 112–114 °C; IR (KBr) 3121 (NH), 2203 (C≡N), 1674 (C = O) cm^−1^; ^1^H NMR (400 MHz, DMSO-d_6_) *δ*: 3.82 (*s*, 3H, OCH_3_), 3.87 (*s*, 3H, OCH_3_), 5.45 (*s*, 1H, NH, D_2_O exchangeable), 7.03 (d, *J=* 8 Hz, 1H, Ar-H, dimethoxyphenyl H-5), 7.10 (d, *J* = 8 Hz, 2H, Ar-H, phenyl H-2, H-6), 7.38 (d, *J=* 8 Hz, dimethoxyphenyl H-6), 7.53 (*s*, 1H, dimethoxyphenyl H-2), 7.68 (d, *J* = 15.6 Hz, 1H, COCH = CH), 7.81 (d, *J* = 15.6, 1H, COCH = CH), 8.19 (d, *J* = 8 Hz, 2H, Ar-H, phenyl H-3, H-5); ^13 ^C NMR (100 MHz, DMSO-d_6_) *δ*: 56.04 (OCH_3_), 56.16 (OCH_3_), 111.03 (dimethoxyphenyl C-2), 112.00 (dimethoxyphenyl C-5), 112.15 (C≡N), 115.43 (phenyl C-3, C-5), 119.79 (CO-*C*H = CH), 124.34 (dimethoxyphenyl C-6), 127.94 (dimethoxyphenyl C-1), 131.27 (phenyl C-2, C-6), 132.60 (phenyl C-1), 143.99 (phenyl C-4), 144.59 (CO-CH = *C*H), 149.42 (dimethoxyphenyl C-4), 151.65 (dimethoxyphenyl C-3), 187.82 (C = O); EIMS (*m*/*z*) 308 (*M*^+^**^.^**, 11.81%), 120 (100%). Anal. Calcd for C_18_H_16_N_2_O_3_: C, 70.12; H, 5.23; N, 9.09. Found: C, 70.09; H, 5.16; N, 9.11.

#### (*ZE*)-*N*-{4-[3–(3,4,5-Trimethoxyphenyl)acryloyl]phenyl}cyanamide (7b)

Brown solid; (1.28 g, 38%) yield; mp 109–111 °C; IR (KBr) 3210 (NH), 2257 (C≡N), 1682 (C = O)cm^−1^; ^1^H NMR (400 MHz, DMSO-d_6_) *δ*: 3.72 (*s*, 3H, *p*-OCH_3_), 3.87 (*s*, 6H, 2 *m*-OCH_3_), 5.40 (*s*, 1H, NH, D_2_O exchangeable), 7.03 (d, *J* = 8.8 Hz, 2H, phenyl H-2, H-6), 7.22 (*s*, 2H, trimethoxyphenyl H-2, H-6), 7.66 (d, *J* = 15.6 Hz, 1H, COCH = CH), 7.86 (d, *J* = 15.6, 1H, COCH = CH), 8.15 (d, *J* = 8.8 Hz, 2H, phenyl H-3, H-5); ^13 ^C NMR (100 MHz, DMSO-d_6_) *δ*: 56.37 (2 *m*-OCH_3_), 60.62 (*p*-OCH_3_), 106.83 (trimethoxyphenyl C-2, C-6), 113.61 (C≡N), 115.78 (phenyl C-3, C-5), 121.62 (CO-*C*H = CH), 130.76 (trimethoxyphenyl C-1), 130.95 (phenyl C-2, C-6), 131.50 (phenyl C-1), 140.03 (trimethoxy C-4), 144.12 (CO-CH = *C*H), 146.52 (phenyl C-4), 153.56 (trimethoxyphenyl (C-3, C-5), 187.48 (C = O); EIMS (*m*/*z*) 338 (*M*^+^**^.^**, 4.68%), 43 (100%). Anal. Calcd for C_19_H_18_N_2_O_4_: C, 67.44; H, 5.36; N, 8.28. Found: C, 67.35; H, 5.27; N, 8.09.

### General procedure for preparation of 1,3,5-triaryl-4,5-dihydro-1*H*-pyrazoles 8a and b

To a solution of the appropriate chalcone **3a** and **b** (0.01 mol) in absolute ethanol (20 mL), 4-methanesulphonylphenylhydrazine hydrochloride (0.33 g, 0.015 mol) was added and the reaction mixture was heated under reflux for 12 h. After completion of the reaction (monitored by TLC plates using chloroform/methanol 9.5:0.5 V/V), the obtained solid was filtered, dried and crystallised from 95% ethanol to give the respective triarylpyrazoles **8a** and **b**.

#### 1-{4-[5-(3,4-Dimethoxyphenyl)-1-(4-(methanesulphonyl)phenyl]-4,5-dihydro-1H-pyrazol-3-yl)phenyl}-1H-tetrazole (8a)

Red solid; (2.01 g, 40%) yield; mp 201–203 C; IR (KBr) 2925–2837 (CH aliphatic), 1294, 1136 (SO_2_) cm^−1^; ^1^H NMR (400 MHz, DMSO-d_6_) *δ*: 3.09 (*s*, 3H, SO_2_CH_3_), 3.29 (dd, *J* = 17.6, 5.6 Hz, 1H, pyrazoline H-4), 3.64 (*s*, 3H, OCH_3_), 3.70 (*s*, 3H, OCH_3_), 4.01(dd, *J* = 17.6, 12.4 Hz, 1H, pyrazoline H′-4), 5.63 (dd, *J* = 12.4, 5.6 Hz, 1H, pyrazoline H-5), 6.74 (d, *J* = 8.4 Hz, 1H, dimethoxyphenyl H-6), 6.91 (d, *J=* 8.4 Hz, 1H, dimethoxyphenyl H-5), 6.98 (*s*, 1H, dimethoxyphenyl H-2), 7.21 (d, *J* = 8.8 Hz, 2H, methanesulphonylphenyl H-3, H-5), 7.71 (d, *J* = 8.8 Hz, 2H, methanesulphonylphenyl H-2, H-6), 8.01–8.10 (*m*, 4H, phenyl H-2, H-3, H-5, H-6), 10.18 (*s*, 1H, tetrazole H); ^13 ^C NMR (100 MHz, DMSO-d_6_) *δ*: 43.45 (pyrazoline C-4), 44.58 (SO_2_CH_3_), 55.96 (2OCH_3_), 63.05 (pyrazoline C-5), 110.16 (dimethoxyphenyl C-2), 112.67 (methanesulphonylphenyl C-2, C-6), 112.99 (dimethoxyphenyl C-6), 117.90 (dimethoxyphenyl C-5), 121.69 (phenyl C-3, C-5), 128.13 (methanesulphonylphenyl C-3, C-5), 129.00 (phenyl C-2, C-6), 129.92 (methylsulphonyl phenyl C-4), 133.29 (phenyl C-4), 134.09 (phenyl C-1), 134.34 (dimethoxyphenyl C-1), 142.62 (tetrazole C-5), 147.50 (dimethoxyphenyl C-4), 148.70 (dimethoxyphenyl C-3), 149.58 (methylsulphonyl phenyl C-1), 149.75 (pyrazole C-3); Anal. Calcd for C_25_H_24_N_6_O_4_S: C, 59.51; H, 4.79; N, 16.66. Found: C, 59.71; H, 4.85; N, 16.74.

#### 1-{4-[1-(4-Methanesulphonyl)phenyl]-5-(3,4,5-trimethoxyphenyl)-4,5-dihydro-1H-pyrazol-3-yl)phenyl)-1H-tetrazole (8b)

Orange solid; (2.24 g, 42%) yield; mp 241–243 °C; IR (KBr) 2924–2855 (CH aliphatic), 1234, 1142 (SO_2_) cm^−1^; ^1^H NMR (400 MHz, DMSO-d_6_) *δ*: 3.10 (*s*, 3H, SO_2_CH_3_), 3.31–3.32 (*m*, 1H, pyrazoline H-4), 3.63 (*s*, 3H, *p*-OCH_3_), 3.71 (*s*, 6H, 2 *m*-OCH_3_), 4.03 (dd, *J* = 18, 12.4 Hz, 1H, pyrazoline H′-4), 5.58 (dd, *J* = 12.4, 6.4 Hz, 1H, pyrazoline H-5), 6.63 (*s*, 2H, trimethoxyphenyl H-2, H-6), 7.23 (d, *J* = 8.8 Hz, 2H, methanesulphonylphenyl H-3, H-5), 7.72 (d, *J* = 8.8 Hz, 2H, methanesulphonylphenyl H-2, H-6), 8.02–8.08 (*m*, 4H, phenyl H-2, H-3, H-5, H-6), 10.19 (*s*, 1H, tetrazole H); ^13 ^C NMR (100 MHz, DMSO-d_6_) *δ*: 43.44 (pyrazoline C-4), 44.54 (SO_2_CH_3_), 56.35 (2 *m*-OCH_3_), 60.46 (*p*-OCH_3_), 63.61 (pyrazoline C-5), 103.29 (trimethoxyphenyl C-2, C-6), 113.06 (methanesulphonylphenyl C-2, C-6), 121.78 (phenyl C-3, C-5), 128.22 (methanesulphonylphenyl C-3, C-5), 129.04 (phenyl C-2, C-6), 129.97 (methylsulphonyl phenyl C-4), 133.22 (phenyl C-4), 134.35 (phenyl C-1), 137.20 (trimethoxyphenyl C-4), 137.65 (trimethoxyphenyl C-1), 142.59 (tetrazole C-5), 147.74 (methylsulphonyl phenyl C-1), 149.94 (pyrazole C-3), 153.85 (trimethoxyphenyl C-3, C-5); EIMS (*m*/*z*) 534 (*M*^+^**^.^**, 0.76%), 43 (100%). Anal. Calcd for C_26_H_26_N_6_O_5_S: C, 58.41; H, 4.90; N, 15.72. Found: C, 58.32; H, 4.86; N, 15.78.

### General procedure for preparation of 1,3,5-triaryl-4,5-dihydro-1H-pyrazoles 9a and b

To a solution of the appropriate chalcone **3a** and **b** (0.01 mol) in absolute ethanol (20 mL), 4-benzenesulphonamidehydrazine hydrochloride (0.33 g, 0.015 mol) was added and the reaction mixture was heated under reflux for 18 h. After completion of the reaction (monitored by TLC plates using chloroform/methanol 9.5:0.5 V/V), the obtained solid was filtered, dried and crystallised from 95% ethanol to give the respective triarylpyrazoles **9a** and **b**.

#### 4-{3-[4-(1H-Tetrazol-1-yl)phenyl]-5-(3,4-dimethoxyphenyl)-4,5-dihydro-1H-pyrazol-1-yl)benzenesulphonamide (9a)

Yellow solid; (2.57 g, 51%) yield; mp 226–228 °C; IR (KBr) 3429 (broad NH_2_), 2929 (CH aliphatic), 1259, 1151 (SO_2_) cm^−1^; ^1^H NMR (400 MHz, DMSO-d_6_) *δ*: 3.30 (dd, *J* = 18, 5.6 Hz, 1H, pyrazoline H**-**4), 3.70 (*s*, 3H, OCH_3_), 3.73 (*s*, 3H, OCH_3_), 3.99 (dd, *J* = 18, 12.4 Hz, 1H, pyrazoline H′**-**4), 5.61 (dd, *J* = 12.4, 5.6 Hz, 1H, pyrazoline H**-**5), 6.64 (d, *J* = 12.8 Hz, 1H, dimethoxyphenyl H-5), 6.99 (d, *J =* 12.8 Hz, 1H, dimethoxyphenyl H-6), 7.00 (*s*, 1H, Ar-H, dimethoxyphenyl H-2), 7.11 (*s*, 2H, NH_2_, D_2_O exchangeable), 7.34 (d, *J* = 8.8 Hz, 2H, benzenesulphonamide H-2, H-6), 7.62 (d, *J* = 8.8 Hz, 2H, benzenesulphonamide H-3, H-5), 8.00–8.16 (*m*, 4H, Ar-H, phenyl H-2, H-3, H-5, H-6), 10.18 (*s*, 1H, tetrazole H); ^13 ^C NMR (100 MHz, DMSO-d_6_) *δ*: 43.66 (pyrazoline C-4), 56.10 (OCH_3_), 56.24 (OCH_3_), 63.12 (pyrazoline C-5), 110.19 (dimethoxyphenyl C-2), 112.82 (benzene sulphonamide C-2, C-6), 117.93 (dimethoxyphenyl C-6), 121.49 (dimethoxyphenyl C-5), 121.68 (phenyl C-3, C-5), 127.31 (phenyl C-2, C-6), 127.98 (benzenesulphonamide C-3, C-5), 130.85 (benzenesulphonamide C-4), 133.49 (phenyl C-4), 133.94 (phenyl C-1), 134.21 (dimethoxyphenyl C-1), 142.64 (tetrazole C-5), 146.27 (benzenesulphonamide C-1), 148.64 (dimethoxyphenyl C-4), 148.87 (dimethoxyphenyl C-3),149.56 (pyrazoline C-3); Anal. Calcd for C_24_H_23_N_7_O_4_S: C, 57.02; H, 4.59; N, 19.39. Found: C, 56.98; H, 4.55; N, 19.22.

#### 4-{3-[4-(1H-Tetrazol-1-yl)phenyl]-5-(3,4,5-trimethoxyphenyl)-4,5-dihydro-1H-pyrazol-1-yl)benzenesulphonamide (9b)

Yellow solid; (2.46 g, 46%) yield; mp 246–248 °C; IR (KBr) 3428 (broad NH_2_), 2940–2839 (CH aliphatic), 1279, 1125 (SO_2_) cm^−1^; ^1^H NMR (400 MHz, DMSO-d_6_) *δ*: 3.32–3.33 (*m*, 1H, pyrazoline H**-**4), 3.71 (*s*, 3H, *p*-OCH_3_), 3.88 (*s*, 6H, 2 *m*-OCH_3_), 4.01–4.03 (*m*, 1H, pyrazoline H′**-**4), 5.57–5.58 (*m*, 1H, pyrazoline H**-**5), 6.61 (*s*, 2H, trimethoxyphenyl H-2, H-6), 7.07–7.18 (*m*, 4H, benzenesulphonamide H-2, H-3, H-5, H-6), 7.64 (*s*, 2H, NH_2_, D_2_O exchangeable), 8.03–8.05 (*m*, 4H, phenyl H-2, H-3, H-5, H-6), 10.18 (*s*, 1H, tetrazole H); ^13 ^C NMR (100 MHz, DMSO-d_6_) *δ*: 40.51 (pyrazoline C-4), 56.35 (OCH_3_), 60.44 (OCH_3_), 63.73 (pyrazoline C-5), 103.32 (dimethoxyphenyl C-2, C-6), 112.85 (benzene sulphonamide C-2, C-6), 121.73 (phenyl C-3, C-5), 127.61 (phenyl C-2, C-6), 128.05 (benzenesulphonamide C-3, C-5), 133.40 (benzenesulphonamide C-4), 134.04 (phenyl C-4), 134.21 (phenyl C-1), 137.16 (dimethoxyphenyl C-1), 137.81 (dimethoxyphenyl C-4), 142.61 (tetrazole C-5), 146.52 (benzenesulphonamide C-1), 149.05 (pyrazoline C-3), 153.82 (dimethoxyphenyl C-3, C-5); EIMS (*m*/*z*) 535 (*M*^+^**^.^**, 0.73%), 43 (100%). Anal. Calcd for C_25_H_25_N_7_O_5_S: C, 56.06; H, 4.70; N, 18.31. Found: C, 55.99; H, 4.75; N, 18.37.

### Biological evaluation

### In vitro cyclooxygenase (COX) inhibition assay

The ability of the test compounds listed in Table 1 to inhibit ovine COX-1 and COX-2 (IC_50_ value, μM) was tested using an enzyme immune assay (EIA) kit (Cayman Chemical, Ann Arbor, MI) was according to a reported method^47^^,^^48^.

**Table 1.** *In vitro* COX-1 and COX-2 inhibition of tested compounds and reference drug, celecoxib.

	IC_50_^a^ (μM)				IC_50_^a^ (μM)		
Compound no.	COX-1	COX-2	SI^b^	Compound no.	COX-1	COX-2	SI^b^
**3a**	12.54	0.57	22	**6a**	9.21	0.38	24.23
**3b**	6.74	0.24	28.08	**6b**	12.89	0.51	25.27
**4a**	6.23	0.43	14.48	**7a**	3.45	0.26	13.26
**4b**	6.11	0.27	22.62	**7b**	3.52	0.24	14.66
**4c**	15.97	0.38	42.02	**8a**	8.67	0.32	27.09
**5a**	7.09	0.18	39.38	**8b**	3.87	0.11	35.18
**5b**	12.41	1.14	10.88	**9a**	7.11	0.33	21.54
**5c**	11.23	0.71	15.81	**9b**	7.37	0.21	35.13
**5d**	11.31	0.45	25.13	**Celecoxib**	7.31	0.16	45.68
**5e**	7.14	0.19	37.57	**Diclofenac**	3.9	0.8	4.87
**5f**	6.45	0.14	46.07	**Indomethacin**	0.039	0.49	0.07

a The *in vitro* test compound concentration required to produce 50% inhibition of COX-1 or COX-2. The result (IC_50_, μM) is the mean of two determinations acquired using an ovine COX-1/COX-2 assay Kit (Catalog No. 560131, Cayman Chemicals Inc., Ann Arbor, MI) and the deviation from the mean is <10% of the mean value.

b The *in vitro* COX-2 selectivity index (COX-1 IC_50_/COX-2 IC_50_).

### In vivo assays

Female wister rats (150–200 g) were used in this study. The animals were kept at controlled conditions (temperature 23 ± 2 °C, humidity 60 ± 10%) and a 12/12-h light/dark cycle with access to food and water. All procedures relating to animal care and treatments were performed according to protocols approved by the Research Ethical Committee of Faculty of Pharmacy Beni-Suef University (2014-Beni-Suef, Egypt).

### Anti-inflammatory activity

The anti-inflammatory activity of the synthesised compounds was determined *in vivo* by Carragenan-induced paw oedema method in rats^49^. Rats were divided into 21 groups of four animals each, and then, they were fasted overnight with free access to water before the experiment. Before any drug administration, thickness of the left hind paw of each rat was measured in millimetres. Group 1 served as a control and administered the vehicle (2.5% Tween 80). Groups (2–20) were orally administered (50 mg/kg) compounds **3a**, **3b**, **4a**, **4b**, **4c**, **5a**, **5b**, **5c**, **5d**, **5e**, **5f**, **6a**, **6b**, **7a**, **7b**, **8a**, **8b**, **9a**, **9b**, respectively. Group 21 was administered celecoxib (50 mg/kg) as a reference standard. paw oedema was induced by subcutaneous injection of 1% carrageen in saline (0.02 mL/rat) into the left hind paw of each rat, one hour after administration of vehicle, test compounds or celecoxib. Paw thickness of each rat was measured after 1, 3, 5 h of Carragenan injection, and then, the change in thickness and % inhibition of paw oedema were calculated.

### Ulcerogenic liability

The ulcerogenic effects of compounds **4a**, **5a**, **5d**, **5e**, **5f**, **8a**, **8b**, **9a**, **9b** and celecoxib were evaluated and compared to that of indomethacin. Forty-eight rats were used in this study, divided into 12 groups and fasted for 18 h before drug administration. The control group received the vehicle (2.5% Tween 80). Other groups received test compounds, celecoxib or indomethacin at a dose of 50 mg/kg, then animals were fed after 2 h. Rats were given the specified dose orally for three successive days. Rats were sacrificed after 2 h of the last dose, then the stomach of each rat was removed and opened along the greater curvature for determination of the ulcer number and ulcer index according to the reported method^50^.

### Analgesic activity

#### Hot plate method

This method was used to evaluate the central analgesic activity by determination of the delay in the latency time of pain response^51^. The analgesic activity of compounds **4a**, **5a**, **5d**, **5e**, **5f**, **8a**, **8b**, **9a**, **9b** and celecoxib were evaluated. The mice were orally administered 10 mg/kg of either test compounds or celecoxib, while 2.5% Tween 80 solution was administered to the normal control group. After 60 min, the animals were placed on a hot plate maintained at 55 ± 0.5 °C. The reaction time was recorded as the time taken by the animals to blow or lick the fore or hind paw or jump off the plate.

#### Writhing method

The assay was performed as described by Koster et al.^52^. Pain was induced by intraperitoneal injection of acetic acid, then counting the number of abdominal writhings. Mice were orally administered 10 mg/kg of compounds **4a**, **5a**, **5d**, **5e**, **5f**, **8a**, **8b**, **9a**, **9b** and celecoxib 30 min before intraperitoneal injection of 0.6% acetic acid. After 5 min, the animals were observed and the number of abdominal writhings was recorded for 20 min.

#### Histopathological investigation

Whole rats stomach were collected, selected stomach samples from each rat were fixed in 10% neutral buffered formalin for 48 h and routinely processed for paraffin embedding. 5 μm Thickness sections were obtained then stained with haematoxylin and eosin for histopathological evaluation^53^.

### Statistical analysis

Significant difference among groups was assessed using one-way ANOVA followed by Dunnett’s test. Differences were considered significant at *p > .05, **p < .01 and ***p > .001. GraphPad Prism software version 5 (Canada) was used to carry out all statistical tests.

### Docking study

Docking was performed to obtain prediction of conformation and also energy ranking between COX-2 receptor (PDB: 1CX2) and the designed compounds^47^. Molecular Operating Environment (MOE, Version 2005.06, Chemical Computing Group Inc., Montreal, Quebec, Canada) was used in docking studies.

The cocrystallised ligand was docked first to study the scoring energy, root mean standard deviation (RMSD) and amino acid interactions. RMSD, for COX-2 enzyme and the lead compound SC-558 was 3 A°.

Docking was performed using London dG force and refinement of the results was done using force field energy. Preparation of the synthesised compounds for docking was achieved *via* their 3 D structure built by MOE. Before docking, 3 D protonation of the structures, running conformational analysis using systemic search and selecting the least energetic conformer were performed. The same docking protocol used with ligand was also applying for the designed compounds. Amino acid interactions and the hydrogen bond lengths were calculated. The data obtained are summarised in Table 4.

## Results and discussion

### Chemistry

**Scheme 1.**
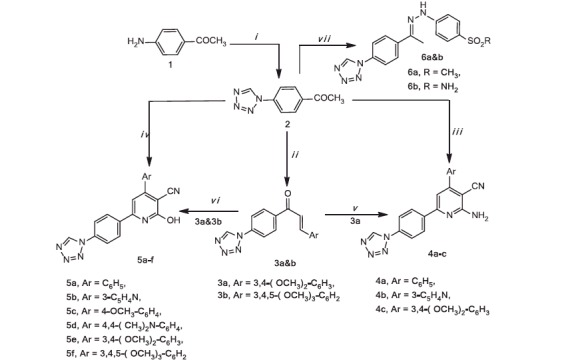
Reagent and conditions: (i) NaN_3_, TEOF, gl. HAc, reflux, 12 h, (ii) ArCHO, KOH, abs. EtOH, r.t., 10–12 h, (iii) ArCHO, CN(CH_2_)CN, NH_4_OAc, abs. EtOH, (iv) ArCHO, CNCH_2_COOEt, NH_4_OAc, abs. EtOH, (v) CN(CH_2_)CN, NH_4_OAc, abs. EtOH, (vi) CNCH_2_COOEt, NH_4_OAc, abs. EtOH, (vii) *p*-substitutedphenylhydrazine hydrochloride, abs. EtOH, reflux, 6–8 h.

**Scheme 2.**
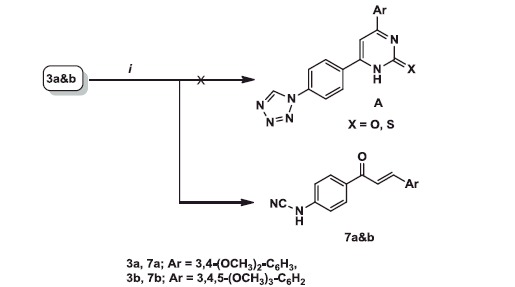
Reagents and conditions: (i) thiourea or urea, KOH, abs. ethanol, reflux 10–12 h.

**Scheme 3.**
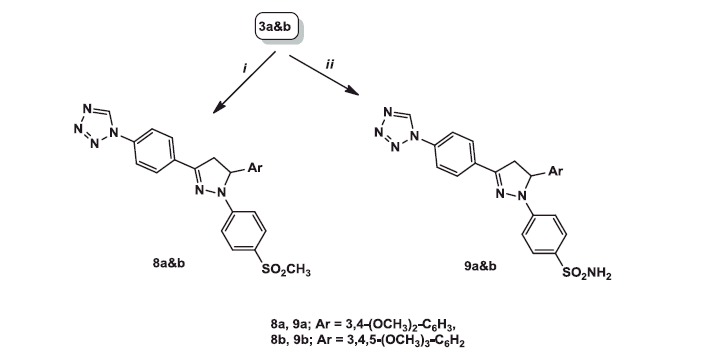
Reagents and conditions: (i) *p*-methanesulphonylphenyl hydrazine hydrochloride, abs. ethanol; (ii) *p*-benzene sulphonamide hydrazine hydrochloride, abs. ethanol.

The synthetic routes of the target compounds are summarised in Schemes 1–3. 1-[4–(1*H*-Tetrazol-1-yl)phenyl]ethanone^2^ was obtained using 4-aminoacetophenone as the starting material according to the literature^54^.

Chalcone derivatives **3a** and **b** were synthesised in high yields (79–86%) by a base catalysed Claisen–Schmidt condensation of acetophenone derivative **2** and substituted aryl aldehydes namely: 3,4-dimethoxybenzaldehyde and 3,4,5-trimethoxybenzaldehyde, respectively.

The prepared compounds have been characterised by IR, ^1^H NMR, ^13 ^C NMR, mass spectra and elemental analyses.

IR spectra of **3a** and **b** showed a sharp peak at 1674, 1663 cm^−1^ corresponding to C = O group. ^1^H NMR spectra of **3a** and **b** revealed the presence of two doublet protons at *δ* 7.77 due to COC***H* **=** **CH and at *δ* 7.90, 7.95 corresponding to COCH = C***H*** proton with high *J* value (15.2 Hz). ^13 ^C NMR spectra of **3a** and **b** showed a peak at *δ* 188.49, 188.60 attributed to C of C = O.

Formation of aminocyanopyridine derivatives **4a–c** and their isosteric hydroxycyanoypyridine derivatives **5a–f** was achieved using two different methods (A and B). In method A, one-pot multicomponent synthetic approach was utilised. Thus, compound **2** reacted with the respective aromatic aldehyde, malononitrile or ethyl cyanoacetate in the presence of ammonium acetate to yield the respective derivatives **4a–c** (45–61%, yield) and **5a–f** (41–57%, yield).

The second procedure (B), stepwise reaction was applied, thus reaction of acetophenone derivative **2** with the respective aldehyde to give the corresponding chalcone **3a** and **b**, followed by the reaction of ammonium acetate and malononitrile or ethyl cyanoacetate to afford **4c** (39%, yield) and **5e** and **f** (41–46%, yield).

Better yield and operational simplicity of the first method (A) encouraged us to prepare the remaining derivatives **4a** and **b** and **5a–d** using it.

IR spectra of cyanopyridine derivatives **4a–c** and **5a–f** showed the appearance of sharp peak at 2226–2207 cm^−1^ corresponding to C≡N group. In addition to a forked peak at 3352–3210 and 3136–3117 cm^−1^ due to NH_2_ in **4a–c**, and a broad peak at 3483–3325 cm^−1^ attributed to OH group in case of **5a–f**. Moreover, the absence of peak due to C = O group of the parents **2** and **3a** and **b** confirmed the structure of **4a–c** and **5a–f**.

The ^1^H NMR spectra of **4a–c** and **5a–f** showed a singlet of one-proton intensity at *δ* 6.84–7.88 corresponding to pyridine H-5. Also, D_2_O exchangeable singlet peak at *δ* 6.99, 7.66 attributed to NH_2_ protons for the aminopyridine derivatives **4a–c**, and a broad exchangeable peak at *δ* 12.65–12.94 corresponding to OH in case of hydroxypyridine derivatives **5a–f**.

^13 ^C NMR spectra of **4a–c** and **5a–f** confirmed the formation of pyridine ring by the presence of three peaks at *δ* 90.87–97.20, 100.91–119.05 and 114.64–117.87 corresponding to pyridine C-3, C-5 and C of C≡N. No evidence for the presence of C = O of the starting compounds **2** and **3a** and **b**.

Condensation of compound **2** with 4-methanesulphonylphenyl hydrazine hydrochloride or 4-benzenesulphonamide hydrazine hydrochloride afforded **6a** and **b**, respectively.

IR spectra of **6a** and **b** showed two sharp peaks at 1277, 1273 and 1157, 1126 cm^−1^ corresponding to SO_2_, in addition to a peak at 3449 and 3440 cm^−1^ due to NH group. There was no evidence for the presence of C = O group that present in the parent compound **2**.

^1^H NMR spectra of **6a** and **b** revealed the presence of an exchangeable singlet signal at *δ* 9.90, 10.05 attributed to NH proton. Another D_2_O exchangeable singlet signal of two protons intensity appeared at *δ* 7.11 corresponding to SO_2_NH_2_ protons in **6b**. Moreover, three protons of SO_2_CH_3_ group in **6a** appeared as a singlet signal at *δ* 3.13.

In an attempt to synthesise pyrimidine derivatives **A** from the reaction of **3a** and **b** with urea or thiourea under reflux temperature in alcoholic KOH, the unexpected chalcone derivatives **7a** and **b** were obtained instead. The deviation of the reaction caused as a result of two factors, heating and presence of a base. Thus, tetrazole ring cleaved with the loss of nitrogen (N_2_) and forming products bearing cyanamide (-NHCN) group. The same explanation for tetrazole cleavage was published by Vorobiov et al.^55^ upon using more drastic conditions (NaOH in DMSO), (Scheme 2).

IR spectra of **7a** and **b** showed three sharp peaks at 3210, 3121; 2257, 2203 and 1682, 1674 cm^−1^ corresponding to NH, C≡N and C = O groups.

^1^H NMR spectra of **7a** and **b** revealed the presence of two doublet signals at *δ* 7.66, 7.68 and 7.81, 7.86 due to COC***H* **=** **CH and COCH = C***H*** protons, respectively with high *J* value (15.6 Hz). On the other hand, absence of signals for both pyrimidine H-5 and tetrazole H-5 confirmed formation of the unexpected products **7a** and **b**.

^13 ^C NMR spectra of **7a** and **b** showed signals for C≡N and C = O at *δ* 112.15, 113.61 and 187.48, 187.82, respectively. No evidence for tetrazole C-5 which present in the parent compounds **3a** and **b** and in compound **A**.

Mass spectra of **7a** and **b** showed molecular ion peak at *m/z* 308 (11.81%) and 338 (4.68%), sequentially.

Another two groups of triarylheterocycles **8a** and **b** and **9a** and **b** were synthesised using the reaction sequence adopted in Scheme 3. Accordingly, condensation of α,β-unsaturated ketones **3a** and **b** with 4-methanesulphonylphenylhydrazine hydrochloride or 4-benzenesulphonamidehydrazine hydrochloride in absolute ethanol gave the respective 1,3,5-triaryl-pyrazolines **8a** and **b** and **9a** and **b**, in good yields (40–51%).

IR spectra of **8a** and **b** and **9a** and **b** showed two sharp peaks at 1294–1234 and 1151–1125 cm^−1^ corresponding to SO_2_. Another broad peak appeared at 3429, 3428 cm^−1^ observed in case of **9a**&**b** due to NH_2_.

^1^H NMR spectra of pyrazolines **8a** and **b** and **9a** and **b** displayed three doublet of doublet (dd) signals each of one-proton intensity at *δ* 3.29–3.33, 3.99–4.03 and 5.57–5.63 with three different *J* values corresponding to three protons of pyrazoline ring. The highest *J* value 17.6–18 Hz was due to germinal coupling of the two protons at C-4 of pyrazoline ring, while the other two *J* values 12.4 and 5.6–6.4 Hz due to coupling of the two geminal protons at C-4 and the vicinal proton at C-5. Additionally, ^1^H NMR spectra of **8a** and **b** revealed the presence of a singlet signal at *δ* 3.09–3.10 due to SO_2_CH_3_ protons. Moreover, an exchangeable signal at *δ* 7.11, 7.64 corresponding to SO_2_NH_2_ protons was observed in ^1^H NMR spectra of **9a** and **b**.

^13 ^C NMR spectra of **8a** and **b** and **9a** and **b** showed two peaks at *δ* 40.51–43.66 and 63.05–63.73 corresponding to C-4 and C-5 of pyrazoline ring.

## Biological evaluation

### Anti-inflammatory activity

#### *In vitro* cyclooxygenase (COX) inhibition assay

The peroxidase activity of cyclooxygenase enzyme isoforms was measured using the COX activity assay kit. The ability of the test compounds to inhibit both ovine COX-1 and COX-2 enzymes was explored *in vitro* using a colorimetric enzyme immunoassay (EIA) kit. The appearance of oxidised *N*,*N*,*N′*,*N′*-tetramethyl-*p*-phenylenediamine (TMPD) was monitored at 590 nm. The kit includes isozyme-specific inhibitors for distinguishing COX-2 activity from that of COX-1. The advantages of this COX assay method are screening a vast number of inhibitors and saving much time.

The *in vitro* test compound concentration required to produce 50% inhibition of COX-1 or COX-2 (IC_50_%) was measured. Moreover, the COX-2 selectivity index (S.I.) values [IC_50_ (COX-1)/IC_50_ (COX-2)] were calculated and compared with that of the standard drug celecoxib (as a selective COX-2 inhibitor), diclofenac (non-selective COX inhibitor) and indomethacin (selective COX-1 inhibitor). All the synthesised compounds were tested; data are listed in Table 1.

The results showed that eight compounds (**3a**, **4c**, **4b**, **5b**, **5c**, **5d**, **6a**, **6b** and **8a)** could inhibit COX-1 at higher dose range with IC_50_ (8.67–15.97 μM) which is more than celecoxib and diclofenac (7.31 and 3.9 μM, respectively). While, compounds (**3b**, **4a**, **4b**, **5a**, **5e**, **5f**, **9a**, **9b** and **8b)** had closed IC_50_ to celecoxib or diclofenac in a range of 3.87–7.37 μM and were less potent than indomethacin (IC_50_ = 0.039 μM). From this point, it was expected that most of the synthesised compounds might be safe with low ulcerogenic effect on gastric mucosa. Regarding to COX-2 inhibitory activity, both trimethoxyphenylpyrazoline derivatives bearing –SO_2_Me pharmacophore **8b** and trimethoxyphenyl pyridine hydroxycarbonitrile analog **5f** had the highest COX-2 potency (IC_50_ = 0.11 and 0.14 μM, respectively), while, IC_50_ of the reference drug celecoxib was 0.16 μM.

Hydroxypyridine carbonitrile analogs **5a** and **5e** exhibited appreciable COX-2 inhibitory activity IC_50_ = 0.18 and 0.19 μM, sequentially, which are closer to celecoxib. Compounds **3a**, **3b**, **4a**, **4b**, **4c**, **5c**, **5d**, **6a, 6b**, **7a, 7b 8a**, **9a** and **9b** (IC_50_ = 0.21–0.71 μM, range) were less potent as COX-2 inhibitors than celecoxib and at the same time, more potent if compared to diclofenac. Pyridopyridine hydroxycarbonitrile derivative **5b** showed the lowest COX-2 potency (IC_50_ = 1.14 μM).

Accordingly, the results showed COX-2 selectivity indices in the range 10.88–46.07. Within the synthesised compounds, compound **5f** had better COX-2 selectivity index (SI = 46.07) than that of celecoxib (SI = 45.68). Compound **4c** had selectivity index (SI = 42.02) near to that of celecoxib. Hydroxypyridine carbonitrile derivatives **5a**, **5e** and compounds containing pyrazoline scaffold with trimethoxy phenyl ring and SO_2_Me or SO_2_NH_2_ moiety **8b** and **9b**, respectively, showed good COX-2 selectivity indices ranged from 35.13 to 37.57. While, chalcone derivatives **3a** and **b**, also, **6a** and **b**, **8a**, **9a**, derivatives and aminopyridine carbontrile derivatives **4b, 5d**, showed lower COX-2 selectivity indices (SI = 21.54–28.08) than celecoxib. The smallest COX-2 selectivity index was obtained from **4a** and **5b**.

### Anti-inflammatory activity

*In vivo* anti-inflammatory activity of the tested compounds was performed using carrageenan-induced rat paw oedema method compared to celecoxib as a reference anti-inflammatory drug.

Mean changes in paw oedema thickness of animals pre-treated with the tested compounds and celecoxib after 1, 3 and 5 h from the induction of inflammation were measured and listed in Table 2.

**Table 2.** Results of *in vivo* anti-inflammatory activities of tested compounds using carrageenan-induced rat paw oedema assay.

	Paw thickness changemean ± SEM (%Inhibition of paw oedema)		
Compound	1h	3h	5h
**Control**	1.57 ± 0.09 (0%)	1.67 ± 0.12 (0%)	1.33 ± 0.10 (0%)
**3a**	1.18 ± 0.14 (25%)	1.53 ± 0.09 (9%)	1.18 ± 0.13 (2%)
**3b**	0.73 ± 0.08*** (54%)	1.45 ± 0.18 (13%)	1.33 ± 0.08 (0%)
**4a**	0.78 ± 0.08** (51%)	1.50 ± 0.19 (10%)	1.33 ± 0.05 (0%)
**4b**	1.28 ± 0.10 (19%)	1.53 ± 0.08 (9%)	1.28 ± 0.14 (4%)
**4c**	0.83 ± 0.15** (47%)	0.70 ± 0.11*** (58%)	1.23 ± 0.11 (8%)
**5a**	0.61 ± 0.08*** (61%)	0.63 ± 0.18*** (54%)	0.53 ± 0.09*** (61%)
**5b**	0.98 ± 0.18* (38%)	1.13 ± 0.15 (33%)	1.10 ± 0.15 (17%)
**5c**	0.68 ± 0.09*** (57%)	1.18 ± 0.11 (30%)	1.15 ± 0.05 (14%)
**5d**	0.45 ± 0.10*** (71%)	0.61 ± 0.12***(63%)	0.76 ± 0.15*(43%)
**5e**	0.45 ± 0.16*** (71%)	0.98 ± 0.03*(42%)	0.68 ± 0.08** (49%)
**5f**	0.33 ± 0.09*** (79%)	1.08 ± 0.10 (36%)	0.93 ± 0.15 (30%)
**6a**	1.10 ± 0.23 (30%)	0.98 ± 0.23*(42%)	1.28 ± 0.08 (4%)
**6b**	0.80 ± 0.26** (49%)	1.05 ± 0.09 (37%)	1.18 ± 0.13 (12%)
**7a**	1.20 ± 0.11 (24%)	1.30 ± 0.15 (22%)	1.33 ± 0.08 (0%)
**7b**	1.08 ± 0.11 (32%)	0.95 ± 0.17*(43%)	1.33 ± 0.08 (0%)
**8a**	1.40 ± 0.14*** (75%)	0.89 ± 0.19**(52%)	1.13 ± 0.11 (15%)
**8b**	0.45 ± 0.10*** (71%)	0.88 ± 0.09**(48%)	0.83 ± 0.17 (38%)
**9a**	0.40 ± 0.00*** (75%)	1.03 ± 0.09 (39%)	1.08 ± 0.20 (19%)
**9b**	0.40 ± 0.11*** (75%)	1.00 ± 0.16*(40%)	1.18 ± 0.20 (12%)
**Celecoxib**	0.38 ± 0.03*** (76%)	0.58 ± 0.11*** (66%)	1.08 ± 0.13 (19%)

Values represent mean ± SEM (*n* = 4). Significance levels *p > .05, **p < .01 and ***p > .001 as compared to the control group.

The reference drug, celecoxib showed 76% and 66% inhibitory activity against carrageenan-induced paw oedema after 1 and 3 h, respectively, then the activity decreased to be 19% after 5 h. Most of the tested compounds showed a significant anti-inflammatory activity (*p* < .001) especially after 1 h from administration.

After 1 h, compound **5f** exhibited anti-inflammatory activity (79%) similar to that of celecoxib (76%). This result was in accordance with the *in vitro* COX-2 assay data.

Compounds containing hydroxypyridine carbonitrile core such as **5d** and **5e** and those with SO_2_Me and SO_2_NH_2_ pharmacophores like **8a**, **8b**, **9a**, **9b** showed strong anti-inflammatory activity of about 71–75%. These compounds also exhibited good COX-2 inhibitory activities.

Moreover, compounds **3b**, **4a**, **5a**, **5c** displayed anti-inflammatory activity percentage of about 51–61%.

The lowest anti-inflammatory activity was observed in compounds **3a**, **4b**, **4c**, **5b**, **6a**, **6b**, **7a** and **7b** in a range 19–49%.

After 3 h and 5 h, the anti-inflammatory activity for most of the synthesised compounds was gradually decreased except for phenyl and *N*,*N*-dimethylaminophenyl derivatives of pyridine hydroxycarbonitrile (**5a** and **5d**, respectively) and dimethoxy phenyl pyrazoline derivative **8a** showing duration of action extended to 3 h to **4a–c** be 54%, 63% and 52%, sequentially.

### Ulcerogenic liability

The *in vivo* ulcerogenic liability was evaluated for celecoxib and the most active anti-inflammatory tested compounds **4c**, **5a**, **5d**, **5e**, **5f**, **8a**, **8b**, **9a** and **9b** relative to indomethacin.

The results of ulcerogenic liability (Table 3) revealed that indomethacin caused the most ulcerogenic toxicity with ulcer index (UI: 22.50), whereas both celecoxib and tested compounds exhibited much lower UI between (0.50–2.00).

**Table 3.** Ulcerogenic liability for compounds **4c**, **5a**, **5d-f**, **8a&b** and **9a&b** compared to reference drugs celecoxib and indomethacin.

Compound	Average number of ulcers	Ulcer index^a^
**Control**	0.25	0.25
**4c**	1.75	1.75
**5a**	0.75	1.25
**5d**	1.25	1.50
**5e**	1.00	1.25
**5f**	1.25	1.25
**8a**	1.50	1.50
**8b**	0.75	0.75
**9a**	1.25	2.00
**9b**	0.25	0.50
**Celecoxib**	0.50	0.50
**Indomethacin**	14.25***	22.50***

a The ulcer index is the sum of % incidence, average severity and average number of ulcers after oral administration of the tested compounds or the reference drug with dose equal 50 mg/kg.

**Table 4.** Molecular modelling data for best poses of the designed compounds **3a&b**, **4a-c**, **5a-f**, **6a&b**, **7a&b**, **8a&b**, **9a&b** and **SC-558** during docking in COX-2 (PDB: 1CX2) active site.

	COX-2					COX-2		
Compound no.	Affinity Kcal/mol	Distance (in A°) from main residue		Functional group	Compound no.	Affinity Kcal/mol	Distance (in A°) from main residue	Functional group
**SC-558**	−10.0340	His90Tyr355	2.422.77	SO_2_NH_2_Pyrazole N-2	**5e**	−19.5404	Tyr355Ser530	2.962.96
**3a**	−14.0261	His90Tyr355	2.422.88	C = O3-OMe	**5f**	−22.4323	His90Tyr355Ser530Tyr385	2.532.992.853.32
**3b**	−16.2638	His90Tyr355	2.422.80	C = O3-OMe	**6a**	−10.6634	His90Tyr355	2.662.54
**4a**	−18.7919	His90	2.50	Tetrazole N-2	**6b**	−11.5834	His90Gly354	2.902.64
**4b**	−4.8888	His90	2.51	Tetrazole N-2	**7a**	−5.5894	His90Tyr355Ser530	2.882.473.02
**4c**	−18.5365	His90Tyr385Ser530	2.502.472.98	Tetrazole N-24-OMe4-OMe	**7b**	−5.5429	Ser530	2.73
**5a**	−18.3959	His90	2.51	Tetrazole N-2	**8a**	−12.8979	His90Tyr385	2.363.26
**5b**	−18.1932	His90	2.52	Tetrazole N-2	**8b**	−15.2648	His90	2.39
**5c**	−12.5497	His90Tyr355	2.543.01	Tetrazole N-2Pyridine N	**9a**	−15.1385	His90Tyr385Ser530	2.412.462.83
**5d**	−7.0670	Tyr355Tyr355	2.752.80	OHC≡N	**9b**	−16.9066	His90Ser353Ser530Ser530	2.382.582.862.92

Compounds **9b** and **8b**, bearing pyrazoline core, trimethoxyphenyl part and COX-2 pharmacophores (SO_2_NH_2_ and SO_2_Me), showed equal or close ulcerogenic liability to celecoxib (UI: 0.50 and 0.75, respectively).

The rest of the tested compounds **4c**, **5a**, **5d**–**f**, **8a** and **9a** exhibited lower toxicity than indomethacin (UI: 22.50), where the UIs were in the range of 1.25–2.00.

### Analgesic activity

#### Hot plate method

**Figure 3.**
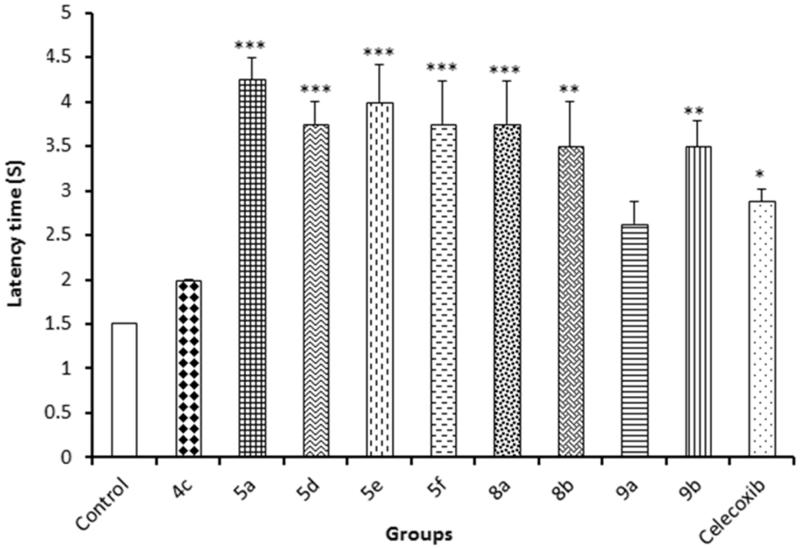
Results of hot plate assay.

This method was used to evaluate the central analgesic activity of nine compounds from the newly synthesised derivatives by determination of the delay in the latency time of pain response. Celecoxib was used as a reference analgesic drug. The delay in the latency time of pain response of the test compounds compared to vehicle-treated animals was determined. All the tested compounds showed potent analgesic activities specially compounds **5e**, **5f**, **8a** and **8b**. These results were consistent with the *in vitro* data on COX, where compounds **5f**, **5e** and **8b** showed COX-2 inhibitory activity with IC_50_ equal to 46.07, 37.57 and 35.18 μM, respectively, (Figure 3).

### Acetic acid-induced writhing test

Peripheral analgesic activity for the tested compounds was investigated using intraperitoneal injection of 0.6% acetic acid after oral administration of mice with 10 mg/kg of compounds **4a**, **5a**, **5d**, **5e**, **5f**, **8a**, **8b**, **9a**, **9b** and celecoxib.

**Figure 4.**
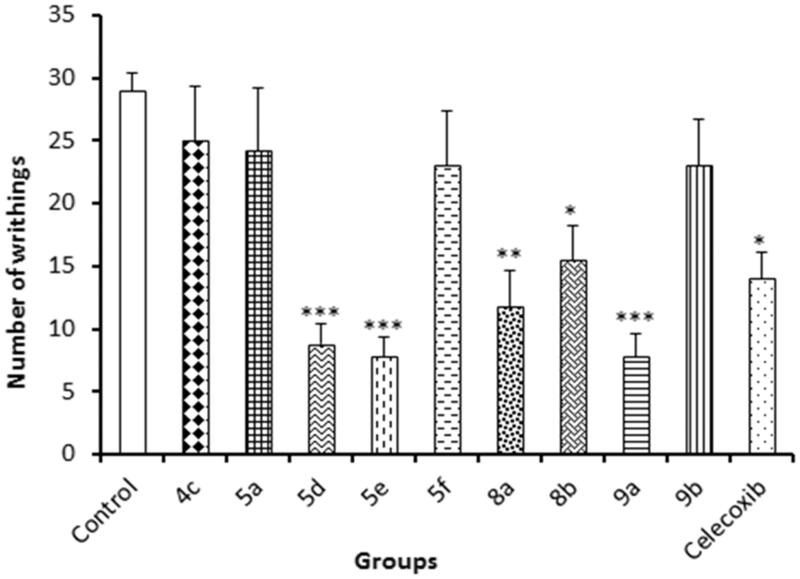
Results of acetic acid-induced writhing test.

The most active compounds were **5d**, **5e** and **9a** compared to normal control. The most potent compound **5e** also has a good inhibitory activity against COX-2 as mentioned previously. The data obtained using this method is summarised in Figure 4.

### Histopathological studies

**Figure 5.**
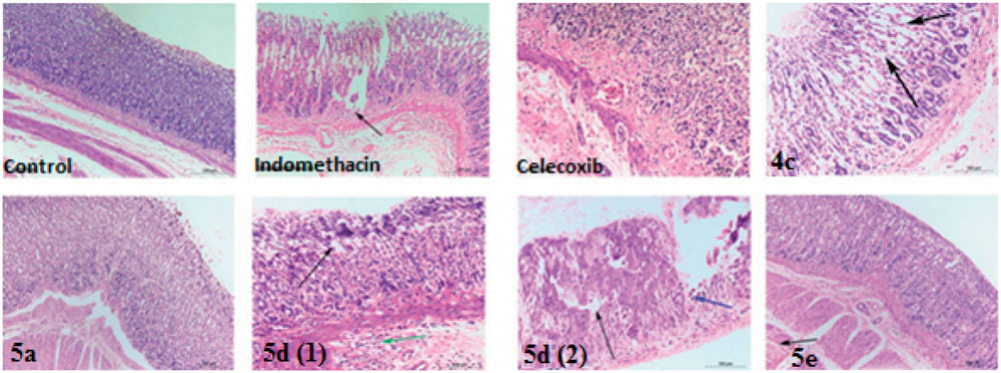
Haematoxylin and eosin immunohistochemical staining of gastric ulcers after ulcer induction in rats for specimen intact Mucous membrane in control, indomethacin, celecoxib-treated rat and test compounds **4c**, **5a**, **5d** and **5e**.

The stomach specimen of control treated rats was characterised by normal histological structure of glandular gastric mucosa, submucosa and musculosa (Figure 5, **control**)^56^. Complete disruption of protective mucosal layer was observed in indomethacin treated rats as mucosa showed an erosion formation (starting point of ulcer formation), also some eosinophilic inflammatory cells infiltration in submucosa associated with hyalinosis and coagulative necrosis of muscular layer and this results in accordance with Günnur et al.^57^ who confirmed that indomethacin induces gastrodoudenal ulcer formation after oral intake (Figure 5, **indomethacin**). There were thickening and hyalinosis of basement membrane with lymphocytic infiltration and minimal congested submucosal blood capillaries after celecoxib intake (Figure 5, **celecoxib**) that is considered much safer drug than indomethacin.

**Figure 6.**
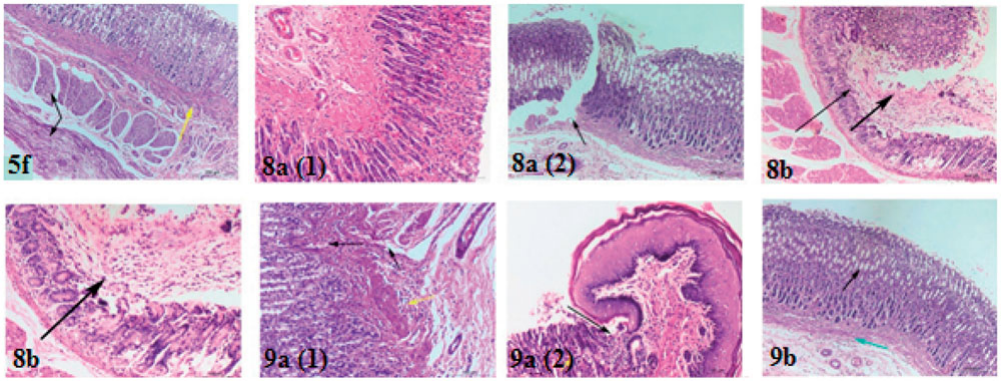
Haematoxylin and eosin immunohistochemical staining of gastric ulcers after ulcer induction in rats for specimen intact mucous membrane in **5f**, **8a**, **8 b**, **9a** and **9 b**-treated rats.

The current tested drug compounds in this study; **4c** treated rats has shown that only lesion on the glandular epithelium of gastric mucosa which showed mucous degeneration in gastric glandular epithelium reach to coagulative necrosis (Figure 5, **4c**). For compound **5a**, all layers of stomach were more or less normal (Figure 5, **5a**). (Ulcer index = 1.5, the short period of oral intake was not enough to induce the tissue reaction towards the drug compound). Mucous degeneration in gastric glandular epithelium, thickening and hyalinosis of basement membrane, as well as eosinophilic infiltrations in submucosa were observed in **5d-**treated rats (Figure 5, **5d** 1, 2). For compounds **5e** (Figure 5, **5e**) and **5f** (Figure 6, **5f**) affect only muscular layer of the stomach by causing corrugation and hyalinosis in some muscle bundles and reach to coagulative necrosis; this changes suggest presence of nausea and irritability of the stomach reach to vomiting may appear as clinical sign in this two groups.

In rats treated with compounds bearing SO_2_CH_3_ pharmacophore and dimethoxy or trimethoxy phenyl part, some areas of glandular epithelium were more or less normal associated with sever congested blood capillaries and eosinophilic infiltrations in submucosa of **8a** treated rats. Ulcer was found in other areas of glandular epithelium of mucosa, thickening and hyalinosis of basement membrane and associated with congestion of blood capillaries, while massive destruction of localised area of epithelium with formation of an erosion associated with hyalinosis and necrosis of muscular layer in **8b** treated rats (Figure 6, **8a** and **b**).

Scanning of stomach specimens of rats treated with compounds **9a** and **9b** (have SO_2_NH_2_ moiety and di- or trimethoxyphenyl group), showed that sever congestion in blood capillaries and lymphocytic infiltration (Figure 6, **9a**, 1). Area of ulceration between glandular epithelium and keratinised epithelium of gastric mucosa and lymphocytic infiltration were noticed. Coagulative necrosis of some cells of glandular epithelium of gastric mucosa (Figure 6, **9a**, 2) were detected. Mucous degeneration of glandular epithelium of gastric mucosa, and lymphocytic infiltration and mild congestion in submucosa **9b** were observed, (Figure 6, **9b**).

### Docking study

All new designed compounds were docked using X-ray crystal structure data for COX-2 enzyme obtained from the protein data bank (pdb: ID 1CX2)^58^.

In docking study, ligand SC-558 and all new designed compounds were docked using Molecular Operating Environment (MOE, Version 2005.06, Chemical Computing Group Inc., Montreal, Quebec, Canada) into the COX-2 receptor.

**Figure 7.**
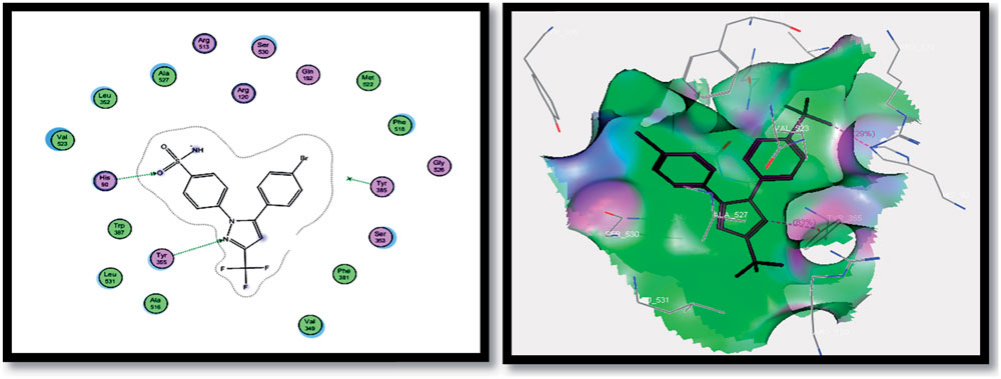
2D (left image) and 3D (right image) interaction of ligand SC-558 in the active site of COX-2 receptor. It is possible to observe the binding using H-bond to His90 and Tyr355 amino acids.

**Figure 8.**
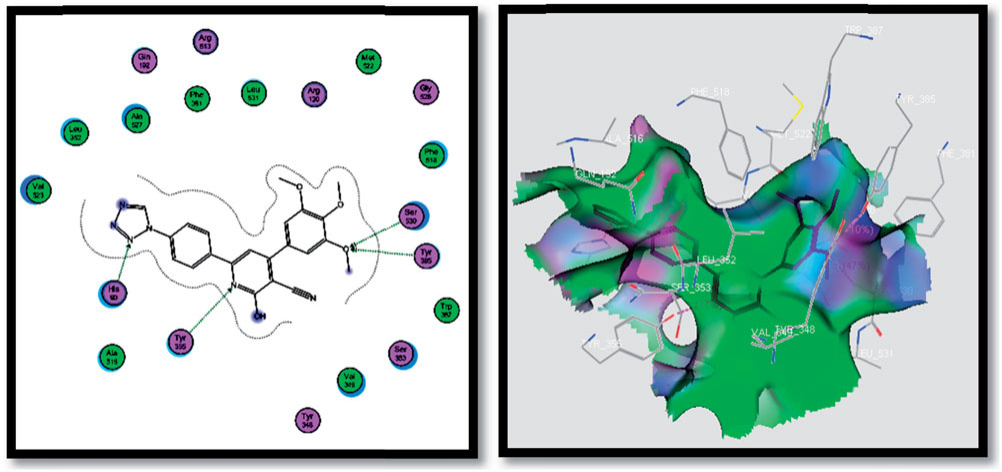
2D (left image) and 3D (right image) interaction of compound **5f** in the active site of COX-2 receptor. It is possible to observe the binding using H-bond to His90, Tyr355, Tyr385 and Ser530 amino acids.

It was observed that H-bonding interactions between ligand **SC-558** (selective COX-2 inhibitor) and COX-2 receptor were achieved *via* two H-bonds (i) SO_2_NH_2_ with His90 (2.42 A°) and (ii) pyrazole N-2 with Tyr355 (2.77 A°). The energy associated with intermolecular interaction was −10.0340 Kcal/mol, Table 4 and Figures 7 and 8.

Most of the designed compounds had the same pattern of interactions in the COX-2 receptor through His90, Tyr355, Tyr385, Ser530, Gly354 and Ser353 amino acids, with one to four H-bonds.

Thirteen compounds **3a** and **b**, **4a** and **c**, **5a–f**, **6a** and **b**, **8a** and **b** and **9a** and **b** showed appreciable binding interactions (affinity in Kcal/mol ranges from −11.5834 to −22.4323) with one to four H-bonding interactions.

Compounds **4b**, **5d**, **7a** and **b** exhibited lower binding interactions than ligand and their affinity range was from −3.6634 to −7.0670 Kcal/mol with different numbers of H-bonds from one to four.

Docking compound **5f** (with COX-2 S.I.=46.07) in the COX-2 enzyme, four H-bonding interactions with His90, Tyr355, Tyr385 and Ser530 amino acids were observed. Its binding energy was −22.4323 Kcal/mol more than that of **SC-558**. Compounds **6a** and **b**, **8a** and **b** and **9a** and **b**, containing COX-2 pharmacophores (SO_2_Me or SO_2_NH_2_), were in high response to COX-2.

On the other hand, affinity of some pyridine containing compounds such as **4b** and **5d**, and the two cyanamide derivatives **7a** and **b** (lack from tetrazole moiety), was lower than that of **SC-588**.

In most compounds, tetrazole N-2 was important for formation of H-bonding interactions with His90 and Tyr355 amino acids, (Table 4).

## Conclusions

In summary, the design and synthesis of new tetrazole **3a** and **b**, **4a–c**, **5a–f**, **8a** and **b** and **9a** and **b** and cyanamide derivatives **7a** and **b** as anti-inflammatory agents were described. *In vitro* COX-1 and COX-2 assay was evaluated. Compound **5f** possessing hydroxypyridine carbonitrile core and bearing trimethoxyphenyl group, was the most potent and selective COX-2 inhibitor with IC_50_ value of 46.07, more than that of celecoxib (IC_50_=45.68). Compounds with pyridine ring **4c**, **5a**, **5e** and those with trimethoxyphenyl ring and pyrazoline core-bearing SO_2_CH_3_ or SO_2_NH_2_ pharmacophores **8b** and **9b** showed high activity as COX-2 inhibitors with IC_50_ ranged from 35.13 to 42.02. All test compounds were evaluated for their *in vivo* anti-inflammatory activities. Analgesic activity (central and peripheral) and ulcerogenic liability were assessed. Compounds **9b** and **8b** with trimethoxyphenyl part and SO_2_NH_2_ or SO_2_CH_3_ pharmacophore have the same or near safety profile as the selective COX-2 inhibitor celecoxib (UI = 0.5, 0.75, respectively). The rest of the tested compounds **4c**, **5a**, **5d–f**, **8a** and **9a** showed remarkable improvement in ulcer index (UI = 1.25–2.00) when compared with indomethacin (UI = 22.5). From histobathological investigations, it was shown that **5a** was the most preferable compound with minimal drug effect on tissue. While **9a** caused destructive effect on the mucosa and ulceration.

Molecular docking study on COX-2-active site showed that in most compounds, tetrazole N-2 was important for the formation of H-bonding interactions with His90 and Tyr355 amino acids similar to SC-558, the ligand used.
